# A review of epigenetic changes in asthma: methylation and acetylation

**DOI:** 10.1186/s13148-021-01049-x

**Published:** 2021-03-29

**Authors:** Mojgan Sheikhpour, Mobina Maleki, Maryam Ebrahimi Vargoorani, Vahid Amiri

**Affiliations:** 1 Department of Mycobacteriology and Pulmonary Research, Pasteur Institute of Iran, Tehran, Iran; 2 Microbiology Research Center, Pasteur Institute of Iran, Tehran, Iran; 3 Department of Microbiology, College of Basic Sciences, Tehran North Branch, Islamic Azad University, Tehran, Iran

**Keywords:** DNA methylation, Acetylation, Epigenetic, Lung diseases, Asthma, Gene expression

## Abstract

Several studies show that childhood and adulthood asthma and its symptoms can be modulated through epigenetic modifications. Epigenetic changes are inheritable modifications that can modify the gene expression without changing the DNA sequence. The most common epigenetic alternations consist of DNA methylation and histone modifications. How these changes lead to asthmatic phenotype or promote the asthma features, in particular by immune pathways regulation, is an understudied topic. Since external effects, like exposure to tobacco smoke, air pollution, and drugs, influence both asthma development and the epigenome, elucidating the role of epigenetic changes in asthma is of great importance. This review presents available evidence on the epigenetic process that drives asthma genes and pathways, with a particular focus on DNA methylation, histone methylation, and acetylation. We gathered and assessed studies conducted in this field over the past two decades. Our study examined asthma in different aspects and also shed light on the limitations and the important factors involved in the outcomes of the studies. To date, most of the studies in this area have been carried out on DNA methylation. Therefore, the need for diagnostic and therapeutic applications through this molecular process calls for more research on the histone modifications in this disease.

## Introduction

**Fig. 1:**
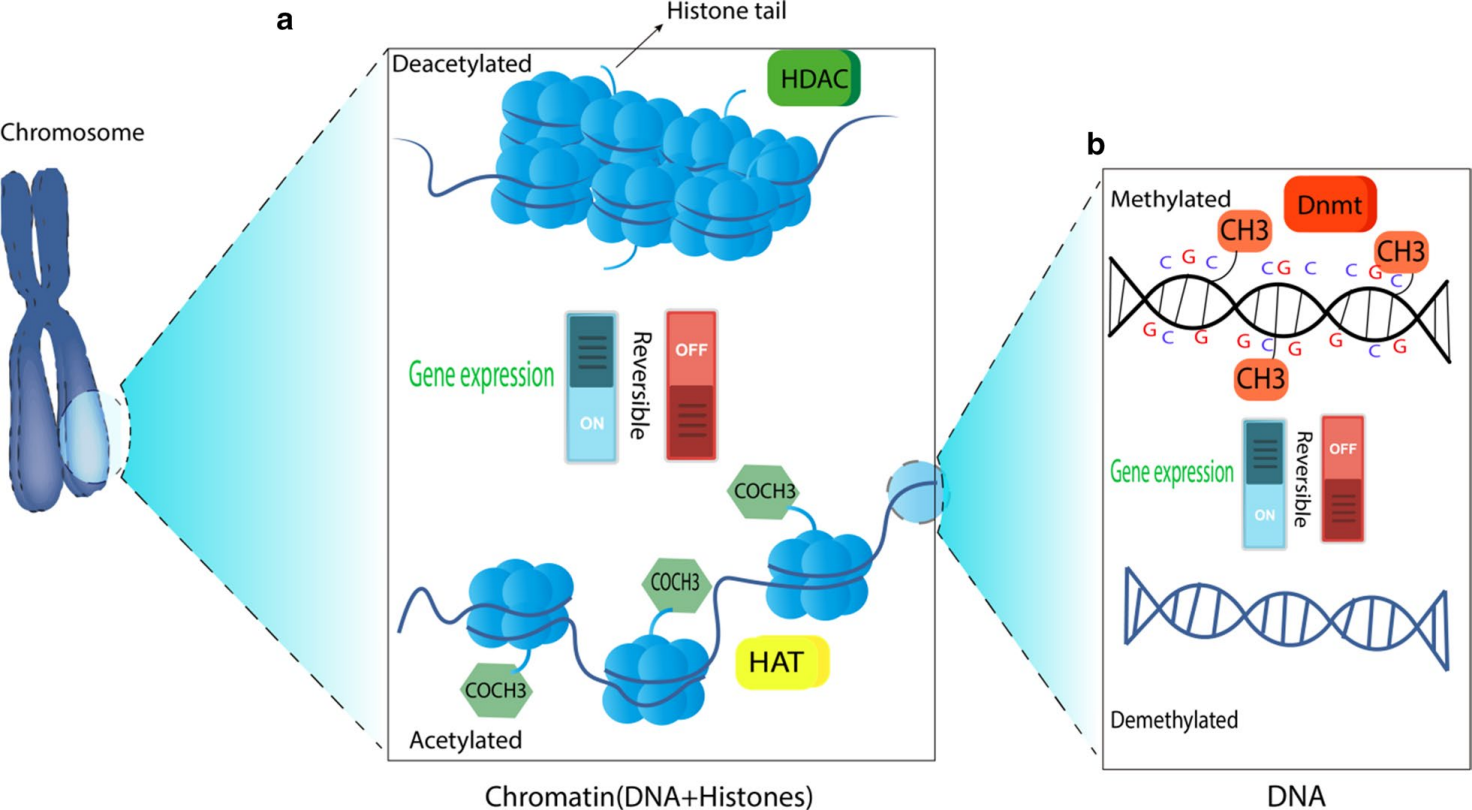
**a** When acetyl groups (COCH3) bind to histone tails via histone acetyltransferase (HAT), the DNA strand around nucleosomes loosens and, as a result, the gene will be expressed. Conversely, the removal of the acetyl groups by histone deacetylase (HDAC) turns off the gene transcription. **b** While the presence of methyl group (CH3) on cytosines inactivates the transcription of a gene, which is catalyzed by DNA methyltransferase (Dnmt), the DNA demethylation contributes to the gene expression

Asthma is a heterogeneous inflammatory airway disease, affecting more than 300 million people globally in a different range of ages by a variable severity. The asthmatic phenotype can be provoked or enhanced by a broad range of triggers, including environmental risk factors, genetic factors, and epigenetic alternations [1–3]. The epigenetic alternation is defined as the chemical modifications applied to the genome deciding whether to express genes or how much of the genome should be expressed in different tissues of the body [4]. Epigenetic alternations, unlike genetic changes, can affect gene expression without changing the DNA sequence. Regular epigenetic modifications consist of DNA methylation, histone modifications, and changes in noncoding RNAs. DNA methylation can silence the genes by the addition of methyl groups to the C5 position of cytosine residues via DNA methyltransferases (Dnmts). This regulatory mechanism can also increase or decrease the expression of genes by hypomethylation and hypermethylation, respectively. Locations on the genome that are reported to have undergone methylation modifications are intergenic regions, gene body, and cytosine-phosphate-guanine (CpG) islands. Although most of the DNA methylation occurs in CpG islands, methylation within gene promoters is essential to the gene silencing process [5]. Gene regulation is also mediated by histone modifications that include histone acetylation, methylation, phosphorylation, and ubiquitination [6]. These post-translational changes can lead to chromatin remodeling and switch the structure between heterochromatin and euchromatin, turning the gene off and on. Two main enzymes in this process are histone acetyltransferases (HATs) and histone deacetylases (HDACs) that have an opposing effect on lysines. While HATs promote gene expression, HDACs have a gene suppressing function [7]. A simple summary of the process is depicted in Fig. 1.

Aberrant epigenetic changes can result in a variety of disorders [8]. Respiratory diseases like asthma are highly affected by inflammatory gene expression which is correlated with epigenetic mechanisms. By the reversible nature of both methylation and acetylation, it is possible to develop drugs and diagnostic tools which target these mechanisms in genes engaged in inflammation as well as other characteristics of the disease [9, 10]. To that end, interrogating the influence of epigenetic changes on genes in different pathways involved in lung diseases such as neurotransmitter pathways can also be taken into consideration [11].

This review will examine the previous epigenetic-based studies on asthma written in the English language. First, we will discuss how asthma affects immune system genes and pathways by exerting methylation and acetylation elements. Then, we will indicate the divergent impact of these changes on distinct subjects. Afterward, the possible extrinsic influences on asthma predisposition through methylation and acetylation, including environmental exposure to tobacco smoke, air pollution, dust mite, and prenatal stress are assessed. Furthermore, during pregnancy, these factors not only affect the mother but also increase the risk of childhood asthma in the next generation [12]. Also, recent studies have shown the undeniable role of genetic and epigenetic interactions through cooperation between single-nucleotide polymorphisms (SNPs), which are DNA sequence variations of individuals, and CpG methylation in regulating gene expression and affecting asthma susceptibility [13]. Finally, conducted experiments on epigenetic differentiation determine how we can benefit more from these studies.

## Airway inflammation in asthma

**Fig. 2:**
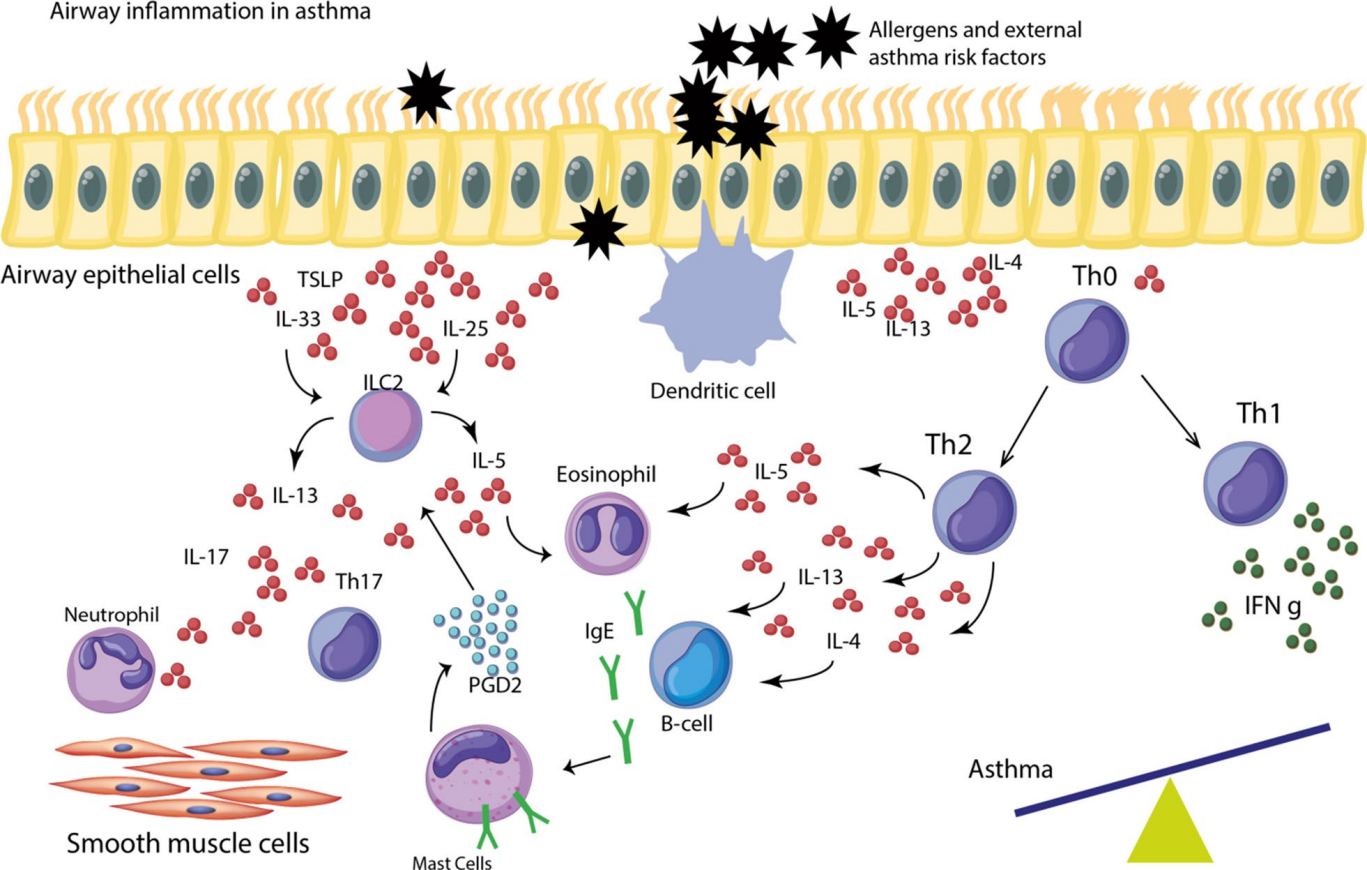
Allergens stimulate structural cells (epithelial cells and smooth muscle cells) to produce different kinds of cytokines, including IL-5, IL-4, IL-13, and thymic stromal lymphopoietin (TSLP). This process activates naïve T-cells (Th0) to differentiate into Th1 and Th2 cells. The imbalance between these T-cells would lead to inflammation in asthma. Released cytokines from Th2 result in IgE production from B-cells, which bind to their receptors on mast cells. Mast cell-derived prostaglandin D2 (PGD2) and Th2 cytokines activate ILC2, which can provoke eosinophilia and hyperactivity in asthma. Th17 and IL-17 are involved in asthma exacerbation by recruitment of neutrophils

A variety of stimuli including air pollution particles, allergens, and viruses can trigger epithelial cells that act as a frontline barrier separating the external environment and the long. As a result of these triggers, airway epithelial cells and smooth muscle cells are activated following which they secrete a plethora of pro-inflammatory mediators such as cytokines (IL-5, IL-4, IL-13, and thymic stromal lymphopoietin (TSLP)), chemokines, and growth factors. In asthma, the interplay of activated dendritic cells (DCs), mast cells, and T helper cells type 2 (Th)2 lead to a perpetual type 2 response that mediates airway inflammatory responses [14]. On the other hand, Th1 cells produce different cytokines, including interferon-gamma (IFN-γ), which can suppress the development of Th2 cells [15]. Th1 and Th2 cells differentiate from naïve T-cells when encountering antigens and the absence of balance in their interaction leads to airway inflammation in asthma [16]. The production of IL-4 and IL-13 by Th2 cells leads to the interaction between T-cells and B-cells, thereby inducing B-cells to produce IgE [17]. Besides, the release of IL-25 and IL-33 from activated epithelial cells together with prostaglandin D2 derived from mast cells activates ILC2. ILC2 is of a pivotal role in promoting type 2 responses in asthma through the production of IL-13 and IL-5, with the former involved in bronchial hyperreactivity and the latter in airway eosinophilia [17, 18]. Asthma exacerbation is governed by the recruitment of neutrophils that is predominantly promoted by Th17 cells and the production of IL-17 [19]. Ultimately, all of these processes contribute to the bronchial hyperreactivity and asthmatic and allergic phenotypes (Fig. 2).

## Asthma-related genes and pathways

Cohorts of asthma patients are diagnosed with high levels of nitric oxide (NO) in their airways [20]. This endogenous product of mammalians has a key role in asthma pathogenesis, which is reviewed in detail by Ghosh and Erzurum [21]. Increased level of NO is the consequence of *induced nitric oxide synthase* (*iNOS*) upregulation at the transcriptional stage, in the bronchial epithelium. Increased level of *iNOS* expression accompanied by eosinophil augmentation in allergic response is associated with the fractional concentration of NO (FeNO) [22]. In the nitric oxide pathway, FeNO is regulated by the expression of different isoforms of arginase (*ARG*) and *NOS*. The promoter methylation of *ARG1* and *ARG2* in buccal cells of asthmatic children was associated with the restrained level of FeNO, whereas there was no link between methylation of *NOS* and the level of FeNO [23]. Nevertheless, Baccarelli et al. [24] reported that high levels of FeNO in asthmatic children were inversely correlated with DNA methylation of *iNOS* and *IL-6* in nasal cells. Although this study could not investigate the effect of methylation on the transcriptional level of these genes, several studies suggest that the methylation change in *iNOS* and *IL-6* promoter leads to their mRNA level regulation [25–27], which can, in turn, affect the airway inflammation in asthmatics. In addition to DNA methylation, dimethylation, and trimethylation of histone H3 lysine 9 in *iNOS* promoter can be involved in its response; this was observed in primary human vascular endothelial cells [25]. Therefore, being able to affect the extent of NO production and the subsequent inflammation in asthmatics, DNA methylation and histone modification at NO pathway genes are a worthwhile target for treatment strategies.

The T helper cells, comprising Th1 and Th2, and their cytokines are differentiated, polarized, and their function is predominantly regulated by the epigenome [28]. In asthma, Th2 cytokines IL-4, IL-13, and IL-5 play a major role in stimulating airway inflammation [29], whereas Th1 cells suppress airway hyperreactivity by producing IFN-γ [30, 31]. Downregulated expression of *IFN-γ* in CD4 + T-cells is found associated with its increased DNA methylation in response to allergic asthma in female mice [32]. On the contrary, a study in 2008 [33] reported a decrease in the methylation degree of the *IFN-γ* and *IL-4* genes and a following increased expression, in CD4 + T-cells isolated from asthmatic patients, after stimulation with different strains of a house dust mite. In the in vivo experiment ran by Verma et al. [34] the expression of *IFN-γ* and *IL-6*, analyzed in mRNA profiling, was significantly low in asthmatic Balb/c mice bronchoalveolar lavage fluid (BALF) cells, trachea, and lung tissues. This low degree was the result of *DNMT1* promoter methylation, a gene on which regulation and development of Th1 and Th2 cells are dependent [34]. Additionally, an increased level of IL-4 and IL-13 in asthmatic primary human bronchial epithelial cells was observed to disrupt the barrier integrity of these cells, which is a defense mechanism in airways. The upregulation of these Th2 cytokines was followed by HDACs 1 and 9 upregulation which was correlated with the defective epithelial barrier [35]. A recent genome-wide study of DNA methylation in nasal epithelium of children with atopy and atopic asthma also has reported several genes associated with Th signaling and epithelium integrity, providing supporting evidence for utilizing methylation status as an asthma diagnostic tool [36]. Therefore, methylation and acetylation on the genes associated with Th cells and the cytokines engaged in inflammation and barrier integrity play a key role in regulating their expression and function in this disease.

Li et al. [37] demonstrate that in peripheral blood of asthmatics, Th2 polarization is inversely correlated with the rate of the linker for activation of T-cells (LAT), Th2 cytokines were significantly upregulated, and *LAT* expression was decreased. This decrement was attributed to hypoacetylation of *LAT* that was observed in lung T-cells of asthmatic rat models. It is notable that trichostatin A treatment in vitro was able to reverse the acetylation level and retrieve *LAT* mRNA expression and thus restrain Th2 cytokine production [37]. The Th1 and Th2 differentiation is also regulated by the enhancers that are specifically methylated in primary T-cells isolated from peripheral blood, varying in healthy subjects and asthmatics. Disease-specific enhancers are prone to acquire dimethylation at histone H3 Lys4 during Th2 differentiation; this is suggested to be involved in asthma pathogenesis [38]. Besides, the suppressor of cytokine signaling-3 (Socs3) regulates the interrelation between Th1 and Th2 cells in asthma [39]. The detected *Socs3* overexpression in asthmatic mice lung tissue is owing to the high levels of histone H4 acetylation at its promoter [40]. This higher level of acetylation, which may lead to tissue remodeling and asthma pathogenesis, was modulated by IL-6. In another study, a high methylation level in the CpG region of *the IL-1 receptor 2* (*IL1R*) 2 gene, which is associated with atopic and non-atopic asthma, was found to be the reason for *IL1R2* low mRNA level in asthmatic individuals' blood [41]. Moreover, in primary bronchial epithelial cell lines derived from asthmatic patients and non-asthmatics, differentiated DNA methylation of *IL-33*, a family member of IL-1, and *CCL26*, an *IL-33* downstream gene, was concordant with asthma status, with the latter showing lower methylation level in response to severe asthma. Nevertheless, despite the positive association found between eosinophil cell count and *CCL26* expression, elevated mRNA level of *CCL26* and *IL-33* in asthmatics was not discriminative between the subjects and controls [42].

In addition to cytokines, the expression of specific chemokines, known as the CXC family, that are responsible for inflammatory cell migration in epithelial cells [43] can also be driven by epigenetic mechanisms. Asthmatics with increased CXCL8, which is a CXC chemokine, secretion in airway smooth muscle cells are prone to promoting steroid-resistant airway inflammation [44, 45]. This upregulation was perceived to be concordant with increased histone H3Lys18 acetylation and an increased number of p300 HATs but not with DNA methylation in primary cultures of human ASM cells isolated from individuals with and without asthma. Moreover, Clifford et al. [44] proposed utilizing BET protein inhibitors as an approach to modify *CXCL8* promoter bindings and thus preventing CXCL8 secretion.

In another study, the HAT loss resulted in the downregulation of the *ORMDL3* gene, which is a childhood asthma gene. Higher levels of accumulated P300 HATs at *ORMDL3* promoter and, therefore, increased acetylation of this gene in lung tissue of asthmatic mice distinguished them from controls [46]. In steroid-resistant asthmatics, defective response to glucocorticoids in bronchial lavage cells stemmed from *the GRβ* overexpression effect on GRβ factors located on the *HDAC2* promoter [47]. To that end, Li and colleagues [47] indicated that by in vitro silencing *glucocorticoid receptor β* (*GRβ*), the decreased expression level of *HDAC2* in bronchial lavage cells could be reverted and increased; besides, this result could not similarly be obtained from peripheral blood mononuclear cells (PBMC). On the contrary, Butler et al. [48] claimed that the level of GRβ was low in people with severe asthma and there was no downregulation affected by GRβ in the level of HDAC1 or HDAC2 in primary bronchial epithelial cells. Notably, the study by Gunawardhana et al. [49] proposed a crosstalk correlation between HDAC and HAT enzyme activities in peripheral blood monocytes of adult neutrophilic asthmatics as increased HAT activity was accompanied by HDAC activity decrement. However, these alternations in the state of activity were not originated from the expression of HDAC1, HDAC2, and other assessed genes. This discordance may be due to the studied tissue type, concerning the fact that epigenetic alternations are tissue-specific [50]. Nevertheless, in an in vivo experiment, Stadhouders et al. [51] have recently demonstrated that subjects with airway inflammation had a similar pattern of histone H3 lys4 dimethylation in group 2 innate lymphoid cells (ILC2s) in different tissues, yet the expression was tissue-specific.

In summary, the interplay between Th1 and Th2, as the promoting factor of inflammation, is driven and disrupted in asthma by epigenetic modifications. Decreased methylation and increased acetylation in Th2 genes lead to an aggravated inflammatory response in asthma. Nevertheless, methylation or acetylation variations in asthma cannot always determine the change in gene expression. Tissue and cell-specific epigenetic interrogations gain better results as epigenetic changes differ in different tissues and also in different cells from the same tissue. Most studies were carried out in vitro, and the most studied tissue was peripheral blood as it was the simplest one to obtain from subjects. Importantly, some studies indicated that the altered methylation and changed expression of some asthma-related genes were not correlated to asthma; therefore, it makes it harder to rely on the results from epigenetic studies that did not measure mRNA expression. Replication of the experiments in another tissue is quite beneficial since it can elucidate the most viable cell or tissue for observing epigenetic regulations in specific genes. Tables 1 and 2 present a brief view of the findings on asthma-related genes and pathways which are reviewed in this chapter.

**Table 1:** Summary of the research works about the DNA methylation role in asthma-related genes and pathways

Author and year	Subjects	Specimen	Genes/regions	Phenotype	References
Kwon et al. 2008	Adults	Venous blood, cultured CD4 +	*IL-4* and *IFN-γ*	Bronchial asthma	[33]
Breton et al. 2011	Children	Buccal cells	*NOS1*, *NOS2A*, *NOS3*, *ARG1*, and *ARG2*	Childhood asthma	[23]
Brand et al. 2012	Female BALB/c mice and BALB/${c}^{\mathrm{scid}}$ mice	Blood	*CNS1* and *IFN-γ*	Asthma	[32]
Baccarelli et al. 2012	Children	Nasal cell	*IL-6* and *iNOS*	Childhood asthma	[24]
Verma et al. 2013	Balb/c mice	lung, trachea tissues and BALF cells and Whole blood	*DNMT1, Socs3, STAT3, IFN-y, IL-4, IL-5 and IL-6*	Asthma	[34]
Gagné-Ouellet et al. 2015	Adult and Children	Whole blood	*IL1R1* and *IL1R2*	Asthma and atopy	[41]
Larouche et al. 2018	Adults	Bronchial epithelial cell line	*CCL26* and *IL-33*	Asthma	[42]
Forno et al. 2019	Children	Nasal cells	30 CpG regions	Atopy and atopic asthma	[36]

**Table 2:** Summary of the research works about the role of histone acetylation and its regulatory enzymes in asthma-related genes and pathways

Author and year	Subjects	Specimen	Gene	Highlighted epigenetic effect	References
Li et al. 2010	Adults	Cultured human A549 alveolar epithelial cells, PBMC and BALF RNA samples	*HDAC2*	HDAC2 is regulated by GRβ resulting in the downregulation of *HDAC2*	[47]
Mishra et al. 2011	Ovalbumin sensitized and challenged mice and saline sensitized and challenged mice	Lung tissue	*Socs3*	Histone H4 acetylation and DNA methylation	[40]
Butler et al. 2012	Adults	Primary bronchial epithelial cell culture	*HDAC2* and *HDAC1*	In severe asthma, no change in HDAC2 and HDAC1 expression caused by GRβ was observed	[48]
Li et al. 2013	Human adults and Wistar rats	Human peripheral blood T-cells and rat lung T-cells	*LAT*	H3 and H4 acetylation and H3 Lys9 dimethylation	[37]
Gunawardhana et al. 2014	Adults	Peripheral blood monocytes and sputum macrophages	-	The interplay between HAT and HDAC affects airway inflammation in asthma	[49]
Seumois et al. 2014	Adults	Peripheral blood and primary human CD4 + T-cells	T-cell enhancers	H3 Lys4 dimethylation	[38]
Clifford et al. 2015	Adults	Primary cultures of human ASM (HASM) cells isolated from bronchial biopsies and large airway tissue	*CXCL8*	H3Lys18 acetylation	[44]
Wawrzyniak et al. 2016	Adults	Air liquid interface cultures of human primary bronchial epithelial cells in	Tight junctions	Histone acetylation	[35]
Stadhouders et al. 2018	Gata3YFP/YFP reporter mice	ILC2s and human peripheral blood	ILC2 genes	H3 lys4 dimethylation	[51]
Cheng et al. 2019	Female BALB/c mice	Lung tissue	*ORMDL3*	Histone acetylation	[46]

## The impact of subject's characteristics

The degree of DNA methylation and asthma susceptibility may vary based on gender type, age of the subject, and certain disorders along with asthma. To that end, Naumova et al. [52] proposed the *ZPBP2* gene in the 17q12-q21 region within which DNA methylation varied not only between male and female asthmatics but also between different ranges of ages. Significance in the differentiation of PBMC DNA methylation status was only observed in male subjects, and the methylation level in adults was higher than in boys. Likewise, in the findings of Reinius et al. [53], adult subjects showed a higher methylation ratio in peripheral blood in comparison with children. Furthermore, interrogating genotype-dependent differentially methylated regions in 5q31 and 17q21 regions led to finding three genes, *GSDMA*, *ZPBP2*, and *SLC22A5* whose promoter methylation was dependent on genotype, sex, and asthma status. Males and females showed a different association between peripheral blood DNA methylation and asthma for each gene [54]. The association between asthma symptoms and the blood spot-derived DNA methylation of *AXL*, a childhood asthma-related gene, was more apparent in girls rather than boys at the same age [55]. On the contrary, Lovinsky-Desir et al. [56] inferred that the methylation level of the *IFN-γ* promoter in PBMC CD4 + lymphocytes and buccal samples was higher in children than adults, and higher in males than females but not in a buccal cell, and with the increase of males' age, the methylation degree decreased. Comparing different tissue types added so much value to their findings as it shed light on the fact that the type of cells can hamper the results derived from an epigenetic study.

**Fig. 3:**
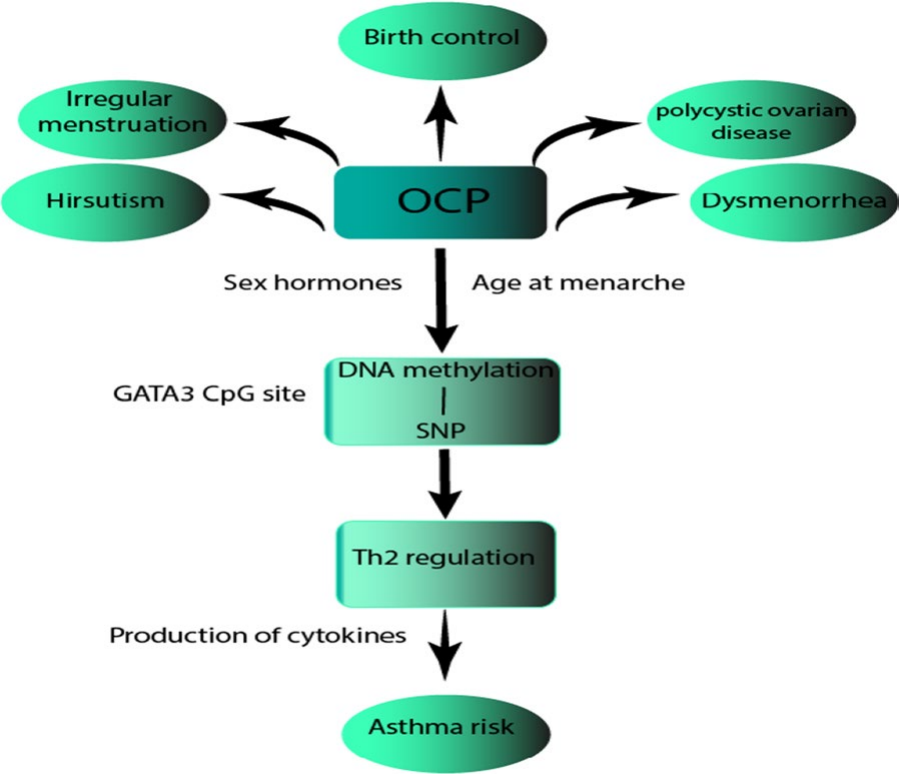
Oral contraceptive pills (OCP), which are used in different conditions, at the age of menarche, can affect *GATA3* methylation level in cytosine-phosphate-guanine (CpG) sites via, presumably, sex hormones. Interaction between *GATA3* single-nucleotide polymorphisms (SNPs) and OCP is influenced by these methylation changes, which affect the immunological process leading to asthma

The DNA methylation alternations throughout the life span are employed as a biological clock. This DNA methylation clock, which is also a biomarker for different diseases including lung cancer [57], may be able to explain age association in allergic diseases [58]. To find the correlation of age with methylation changes and occurrence of asthma, several studies have been done on birth cohort populations following up the subjects before and after birth and investigating their status at certain ages. Employing the Hovath method [59], DNA methylation age was evaluated in three stages of childhood and was considered older than chronological age. In middle childhood (7 to 8 years of age), epigenetic age acceleration was concordant with increased IgE in serum and, accordingly, associated with the risk of allergy and asthma, increasing the odds of these diseases as the methylation age increased [60]**.** Furthermore, Curtin et al. [61] demonstrated the positive correlation between the increased age of participant children (between ages 2–8), augmented methylation of *IL-2* in cord blood, and the risk of asthma exacerbation. Notably, these children were prenatally classified as high and low risk for asthma regarding their parents' atopy status; however, maternal atopy was reported unrelated to *IL-2* methylation at one site and, additionally, atopic children with atopic mothers were of lower methylation in another site of *IL-2* compared with children with non-atopic mothers*.* Differentiated methylation on Th2 pathway genes, *IL4*, *IL4R*, and *GATA3*, in peripheral blood, was distinguishable between female subjects who had asthma at 10 but grew out of it at 18 and the ones healthy at 10 but showed asthma symptoms at 18 [62]. Furthermore, exposure to vaccines and pills in females may affect the epigenetic regulations and thus asthma susceptibility. Another study on a whole population birth cohort reported differentiated DNA methylation patterns in certain CpG sites in the Guthrie cards and whole blood of followed-up female participants who had Tetanus vaccination between the ages 10 and 18. At age 18, this observed altered CpG methylation level was associated with lower odds of asthma susceptibility, so it can be proposed as a preventative approach against developing this disease [63]. In contrast, consuming oral contraceptive pills (OCP) at age 18 was reported to increase the predisposition to asthma by impacting the *GATA3* gene through the SNP-CpG methylation interplay, which was assessed using peripheral blood cells and saliva samples (Fig. 3) [64]. In another study, by investigating the DNA extracted from older smokers 'sputum, Sood et al. [65] found the association of asthma and the methylation of the *protocadherin-20* gene whose methylation degree was emerged to be higher than the healthy group with a similar smoking history. Although this study does not directly address the effect of age on DNA methylation in asthmatic subjects, it provides evidence that as people age the influence of the environment (smoking) increases and may affect the susceptibility to the disease. Yet, this still cannot prove whether asthma was the consequence of an accumulation of methylation during lifetime or asthma inflammation itself led to further methylation of specific genes in these individuals.

In addition to sex and age, there are disorders, like obesity, that can relate epigenetic of asthma to the subject's characteristics. Obesity and high body mass index (BMI) are believed to impact non-atopic asthma [66, 67]. Rastogi and colleagues [68] reported that in PBMCs of obese asthmatic children, genes associated with Th1 polarization and macrophage activation pathway were hypomethylated, whereas genes related to T-cell-mediated inflammation were hypermethylated. These results are consistent with the findings of Jeong et al. [69] that indicated that DNA methylation derived from peripheral blood may be a key link to associate adult non-atopic asthma with BMI status and its change over a while. DNA methylation differentiation in pro-inflammatory pathway genes was considered to mediate the correlation of inflammatory signaling pathways with BMI and NLRP3-IL1B-IL17 axis with BMI shift in 10 years. Another disorder that may influence asthma is Alzheimer's disease. Wu et al. [70], by conducting an in vivo experiment have recently demonstrated that ovalbumin-induced mice with Alzheimer's disease are seen with more regulatory T-cells (*Tregs*) and *forkhead box P3* (*Foxp3*) expression in their lungs, and therefore showing mitigated asthma symptoms compared to the controls. They have suggested that *Foxp3* upregulation may be due to the drop observed in the promoter methylation level of this gene in isolated BALF cells.

These studies highlight the importance of considering gender- and age-specific epigenetic patterns along with the influence of drugs, vaccines, and disorders in asthma. In this section, most of the studies have considered the age factor by using birth cohort samples in their experiments. Despite the limitations in this process, such as only being able to measure the methylation changes at certain time points, these studies have achieved more valuable results as methylation profiles transform throughout a lifetime. Although most of the studies are carried out on children implying the importance of early-life epigenetic modification, the age in which these methylation changes have their highest impact on an individual's asthmatic status is not established yet. According to Hovath and Steve [59], there is a logarithmic association between chronological age and methylation age by 20 years of age but a linear relation after that. Although different aspects should be considered in studying the effects of aging on the methylation status and finally the odds of asthma, aging can be simply seen as more time for the accumulation of asthma risk factors. Of note, the most important thing that was missing in these studies was that not all of them measured the transcriptional level after detecting the methylation changes in asthma-related genes. Peripheral blood was used in almost all of the studies in this section, and the studies which compared peripheral blood and whole blood inferred that whole blood could not fully reflect methylation changes in subjects. Table 3 demonstrates a summary of the articles on the role of characteristics of the subjects who developed asthma on their methylation status.

**Table 3:** Summary of the studies on the impact of the subject's characteristics on the methylation status in asthma

Author and references	Year	Subject	Characteristics	Specimen	Genes/regions
Sood et al. [65]	2012	Old male adults	Age	Sputum	*PAX5-α *and* PCDH20*
Naumova et al. [52]	2013	Male and female adults and children	Sex and age	PBMC	*ZPBP2* and 17q12-q21 region
Reinius et al. [53]	2013	Children and adults	Age	Peripheral blood	*NPSR1*
Rastogi et al. [68]	2013	Children	Disorder: obesity	PBMC	*CCL5,IL2RA,TBX21,FCER2,TGFB1*, Th1 polarization-associated genes, IgE low-affinity receptor gene, and Th cell activation inhibitor gene
Curtin et al. [61]	2013	Children with ages 2 and 8	Age	Cord blood	*IL-2*
Lovinsky-Desir et al. [56]	2014	Adult and children	Age	Buccal cells and PBMC	*IFN-γ*
Zhang et al. [62]	2014	Girls with the age of 10 and 18	Age	Whole and peripheral blood	*IL4, IL4R, IL13, GATA3,* and *STAT6*
Guthikonda et al. [64]	2014	18-year-old females	Drug: OCP	Peripheral blood	*GATA3*
Tuwaijri et al. [54]	2016	Male and female adults	Sex	Peripheral blood	17q12–q21 and 5q31.1 regions
Janjanam et al. [63]	2016	10- and 18-year-old females	Vaccination	Guthrie cards and whole blood	*KIAA1549L, PSMG3*, and *TFAMP1*
Gao et al. [55]	2017	Children	Sex	Bloodspots	*AXL*
Peng et al. [60]	2019	Children	Age	Peripheral and cord blood	-
Jeong et al. [69]	2019	Female adults	BMI status	Peripheral blood	Inflammatory pathway and NLRP3-IL1B-IL17 axis-related genes
Wu et al. [70]	2019	APP/PS1 and C57BL/6 J mice	Disorder: Alzheimer's disease	BALF cells	*Foxp3*

## Environmental exposure

Early-life exposure to asthma risk factors affects childhood and adulthood health and the susceptibility to chronic diseases like asthma [71]. Throughout the number of studies on asthma, environmental factors have proved their influence on the epigenome and gene expression. Therefore, it is of great importance to interrogate how the external factors provoke molecular changes and evaluate the inheritance possibility of the resulting changes.

### Asthma predisposition caused by exposure to cigarette smoke

Asthmatic mothers with different severity of active asthma were able to pass the disease to their offspring, and this may be carried out by epigenetic regulations, regarding their affectability from the environment [72]. In utero exposure to cigarette smoke or secondhand smoke is one of the reasons for lung malfunction and the increase of the asthmatic phenotypes in populations [73, 74]. In an in vivo experiment, sensitized mice with tobacco smoke in utero were exposed to dust mite to develop allergic asthma. Induced allergic asthma led to lower levels of methylation in inflammatory-related genes, comprising IL-4, IL-5, IL-13, and also global DNA methylation in their lung tissue; therefore, more Th2 cytokines were produced resulting in lung inflammation [75]. Since mRNA level was not evaluated in their study and the measurements were not cell-specific but only tissue-specific, increased protein level cannot be attributed merely to DNA methylation changes. Investigating the epigenetic effect of prenatal tobacco smoking on developing childhood asthma, Gao et al. [76] found the extent of *AXL* methylation at birth, measured in bloodspots, associated with maternal smoking and rising asthma symptoms such as wheezing at age 10 in two population study cohorts.

Additionally, Neophytou et al. [77] indicated that the reported exposure to prenatal maternal tobacco smoke in 8- to 21-year-old asthmatic Latino children was associated with higher methylation levels in the *AHHR* gene measured in whole blood. While most studies support the role of maternal smoking in developing asthma in offsprings [74], paternal tobacco smoking can also reprogram the offspring's epigenome. Analysis of cord blood and blood samples were taken right after birth and then at the age of 18 months and 6 years, respectively, showed the role of paternal smoking in higher CpG methylation of *LMO2* and *IL-10* immune genes, and that the extent of methylation was associated with the dose of smoking [78]. Furthermore, Sarnowski et al. [79] indicated that a paternally inherited genetic variant, related to asthma and allergic rhinitis comorbidity, was regulated by differentiated DNA methylation patterns of *MTNR1A* in peripheral blood leukocytes*.* DNA methylation of this gene also mediated the SNP related to asthma and allergic rhinitis comorbidity. Given these points, both parents can be involved in developing asthma in their children through epigenetic alternations driven by exposure to cigarette smoke prenatally and postnatally.

### Exposure to air pollutants and chemical substances

Air pollution-related exposure, in different stages of life, can determine the susceptibility to asthma by epigenetic modulations. Transplacental exposure to traffic-related polycyclic aromatic hydrocarbons was reported to alter the methylation of the *ACSL3* CpG site in umbilical cord white blood cells. This modification was concordant with *ACSL3* expression obtained from matching fetal placental tissue, and thus downregulated the transcription and increased childhood asthma predisposition in children under five [80]. By studying the short-term exposure to PM2.5 and vanadium, Jung et al. [81] inferred that only vanadium, not PM2.5, altered the DNA methylation of *IL4* and *IFN- γ* in buccal samples*,* but this alternation was not concordant with defected lung function. On the contrary, PM2.5 exposures were correlated with the lung function decrement. Differentiation of *FOXP3* and *IL10* DNA methylation was identified in the PBMC of children with asthma who were exposed to NO2, CO, and PM2.5 in the long term (2 years), and the methylation of *FOXP3* promoter augmented because of all three types of pollutants mentioned. Although occurred methylation did not affect the *FOXP3* expression, it was negatively correlated with the activation of Treg cells [82]. Besides, exposing mice to the concurring effect of PM2.5 and cold stress resulted in the decrement of HDAC1 and hyperacetylation of H3K9 and H3K14 in *IL4* promoter in CD4 + cells isolated from BALF. This study also indicates that the exacerbation of asthma is more likely to occur when exposed to various asthma risk factors rather than one [83].

Another set of air pollutants that affect asthma pathway genes epigenetically is black carbon and sulfate. Exposure to these particular pollutants was associated with differentiated DNA methylation of genes related to major histocompatibility complex, eotaxins, ILs, cytokines, eosinophil granule major basic protein, and IgE receptors in peripheral blood. One of the influenced genes for the latter was *FCER1G,* the only one that was affected by both black carbon and sulfate [84]. Exposure to higher levels of black carbon is correlated to lower levels of *IL-4* methylation in children's buccal cells; this methylation level was even lower in children sensitized with cockroach allergens than in non-sensitized children [85]. In vitro analysis of nasal epithelial cells derived from siblings between 5 and 18 years old identified three CpG sites related to childhood asthma none of which witnessed statistically significant methylation change when the cells were exposed to air pollution in asthmatics. Nonetheless, these CpG sites were close to *NLRP3*, *OR2B11*, and *TET-1* genes whose expression was altered in cultured cells as a response to house dust mite and diesel exhaust particles [86]. In line with the previous study, Somineni et al. [87] reported that in nasal airway epithelial cells, higher and lower methylation of a certain CpG site of *TET-1* was associated with exposure to traffic-related air pollutants and asthma in children, respectively.

In addition to air pollutants, there are several toxic environmental substances used in various products that their connection with asthma and methylation marks is investigated. Kuo et al. [88] reported that phthalate is associated with an increased risk of asthma because of promoting Th2 response and compromising *IFN alpha/IFN beta* expression in plasmacytoid dendritic cells isolated from peripheral blood. Phthalate exposure could also attenuate the methylation level of *TNF*-α in whole blood white blood cells and lead to its upregulation, which is an event that increases the risk of asthma in children [89]. The results from another study indicate that exposure to bisphenol A can lead to a decrease in an inflammatory mediator gene *MAPK1* methylation in whole blood, provoking childhood asthma [90]. Studies on inner-city children with persistent atopic asthma suggested that persistence in these children was accompanied by promoting the expression of genes related to Th2 immunity. This upregulation was the consequence of *IL-4*, *IL-13*, and *RUNX3* hypomethylation measured in PBMCs of asthmatic patients compared to the control group [91]. In another study on inner-city children, the results elicited from saliva or whole blood DNA of asthmatics exhibited that increased methylation in the beta-2 adrenergic receptor gene (*ADRB2*) was correlated to decreased dyspnea and asthma severity [92]. Conversely, Fu et al. [93] found that higher levels of the *ADRB2* methylation in blood increase childhood asthma severity. The hypermethylation in *ADRB2* was also suggested to augment the influence of NO2 exposure in asthmatic children and exacerbate the level of asthma [93]. Besides, in a recent study, a positive association between the hypermethylation of this gene and uncontrolled childhood bronchial asthma has been shown. Also, there is a positive correlation between the augmented methylation of *ADRB2* and increased aluminum concentration measured in whole blood [94]. These findings support this hypothesis that there is a synergic relation between environmental asthma risk factors and epigenetic changes.

### Other external influences

Considering race, ethnicity, socioeconomic status, and asthma severity in asthmatic children with low salary families, Chan et al. [95] indicated that higher global methylation of peripheral blood cells was related to African-Americans with persistent asthma whose families had low incomes. Moreover, assessing asthma and/or rhinitis-related CpG regions in African-American and Puerto Rican children led to identifying one site cg03565274 that was significantly methylated and altered the expression of the *ZMYND10* gene in nasal epithelial cells. This region and the majority of other 60 identified CpG sites were correlated with the exposure of children to fury pets at secondary school age [96]. In utero malnutrition has been found as another external factor of increasing experimental asthma risk in offsprings both in vivo and in vitro. Bisulfate sequencing of extracted DNA from naive CD4 + cells isolated from spleens demonstrated increased hypomethylation in Th2 cytokines due to in utero protein restriction diet. As a result, CD4 + T-cell proliferation, activation, and their tendency to differentiate into Th2 cells elevated [97]. Taking selenium and methyl donor nutrients in children with asthma may contribute to alleviating asthma, but it was not clear whether DNA methylation in buccal cells or other epigenetic regulations mediated this process [98]. Furthermore, even seasonal change, as an environmental factor, had an impact on the methylation pattern of *NSPR1* promoter in whole blood, an asthma-related gene during the disease period [53]. According to Torrone et al. [99], the duration of asthma and the short-term environmental change affected *iNOS* and *IFN-γ* methylation degree in buccal cells in a 4- to 7-day period of the disease. This demonstrates the dynamic feasibility of the epigenome related to the asthma genes in response to environmental transformations. In a recent in vitro study, early viral infection with human rhinovirus was also observed to be effective on DNA methylation and then mRNA expression differentiation in asthmatic children's cultured primary nasal epithelial cells [100].

### Probable transgenerational immunity against asthma

Microbial exposure as a preventative approach in allergic diseases is an understudied topic. According to the hygiene hypothesis, being exposed to microbial environments in early life can affect host immunity and reduce the risk of allergic diseases, including asthma [101]. Different factors that are involved in shaping a protective immunity against allergic diseases include delivery mode (cesarean or vaginal), breastfeeding, contacting with a wider range of people, growing up in a rural environment, and probiotics [102]. Although these factors are highly supported by different studies, there are still limitations to assess this matter fully due to the existing biases and contradictory studies. For instance, probiotic bacteria influence the microbiome development in early life and have shown positive effects on atopic dermatitis prevention [103]; however, some studies indicate their opposite effects for asthma [104, 105]. Just like i*n utero* and early exposures to asthma risk factors in early life that may elevate the odds of asthma in childhood and adulthood, being exposed to harmless infections at the same period may have a positive impact against asthma. As the hygiene hypothesis indicated first-year exposure to the farm environment, as opposed to later exposure, was remarkably associated with asthma [101]. In addition to that, the duration of this exposure was taken into account, meaning that children who spent more time on farms (children of full-time farmers) were less susceptible to asthma [106]. Since epigenetic modifications are highly affected by the environment, evaluating the effects of this kind of exposure on the epigenetic process is a viable solution to clarify this phenomenon and contribute to its understanding.

Brand et al. [107] assessed the epigenetic alternation in murine offspring in which prenatal asthma protection was induced by *Acinetobacter lwoffii* F78, a farm-derived gram-negative bacterium. Their results demonstrated that transitional protection altered H4 acetylation and led to forming an asthma preventative phenotype. Michel et al. [108] called into question the effect of farm exposure on the DNA methylation pattern and asthma development in children whom they followed until 4.5 years of age. Their basis of comparisons regarding DNA methylation status was fourfold: asthmatics, non-asthmatics, farmers' children, and non-farmers' children. Within examined ten genes in cord blood, the methylation status of *ORMDL1* and *STAT6* in healthy farmers 'children in comparison with nonfarmers' children with asthma was distinguishable. On the other hand, carrying out the same comparisons for methylation degrees in whole blood samples of these children at 4.5 years of age did not bring out considerable methylation changes between compared groups. In a recent study on the diversity of the gut microbiome, Kyburz et al. [109] exposed female mice to *helicobacter pylori* extract and examined their offsprings for the next two generations. Both F1 and F2 generations have been subjected to house dust mite allergen. As a result of sensitization to the bacterium, in both generations, the susceptibility to airway inflammation was reduced. This reduction was evaluated by observing a drop by 5 to 15 percent in *Foxp3* methylation status after treatment with pylori extract. Studying allergen-specific immunotherapy, Wang et al. [110] investigated the molecular mechanism of this effective therapy in PBMC of asthmatic children who were treated with *Dermatophagoides pteronyssinus* and also in vitro stimulation in cultured PBMCs. Accordingly, increased DNA methylation of *IL-4* promoter and lower release of IL-4, IL-5, and IL-2 were observed, which was put down to decreased sensitization to that specific dust mite. Epigenetic studies may help to identify specific time points when microbiome exposure is of the highest impact as age has a significant effect on epigenetic regulations. However, long-term maintenance of this protection and its effectiveness is yet to be discovered.

To conclude, only a few studies have been conducted on adults and the largest proportion was dedicated to children which included several follow-up studies. The studies on the role of epigenetic modulations in developing asthma protection are very limited and there is still considerable ambiguity in this area that demands to be unraveled in further studies. The most used tissue type was PBMC which can be considered a negative point, although it has a higher value compared with the heterogeneity of cell types in whole blood [111]. Also, common limitations in these studies were lacking ample study cohort size and not being able to carry out replications to evaluate their results. Moreover, although the role of maternal status in the development of asthma is undeniable, there is not enough evidence on the epigenetic mechanisms underlying these effects on offsprings. Both Th1- and Th2-related genes undergo epigenetic modifications that resulted from environmental effects. Exposure to different types of air pollutants or environmental chemicals affects both DNA methylation and histone modifications. Table 4 indicates the reviewed articles in the field of transgenerational effects and environmental exposure.

**Table 4:** Summary of studies on transgenerational effects of epigenetic regulations in asthma

Author and year	Subjects	Gene/region	Epigenetic change	Environment	Phenotype	References
Perera et al. 2009	Dominican and African-American women and their children	*ACSL3*	CpG methylation	Transplacental Exposure to Airborne Polycyclic Aromatic Hydrocarbons	Childhood asthma	[80]
Brand et al. 2011	BALB/c mice and BALB/cscid mice	*IFN-γ, IL-4, IL-5*, and *CNS1*	histone H4 acetylation	Microbiome	Transmaternal asthma protection	[107]
Torrone et al. 2012	Inner-city children	*IFN-γ* and *iNOS*	DNA methylation	-	Asthma	[99]
Fu et al. 2012	Children	*ADRB2*	Hypermethylation	NO2 exposure	Childhood asthma severity	[93]
Reinius et al. 2013	Children and adults	*NPSR1*	DNA methylation	Current smoking in adults and parental smoking in children	Adult severe asthma and childhood allergic asthma	[53]
Kuo et al. 2013	Adults	*IRF7*	Histone H3Lys4 trimethylation	Exposure to Diethylhexyl phthalate and butyl benzyl phthalate	Asthma development	[88]
Michel et al. 2013	Children	*ORMDL1, ORMDL2, ORMDL3, CHI3L1, RAD50, IL13, IL4, STAT6, FOXP3*, and *RUNX3*	DNA methylation	Farm exposure in early childhood	Asthma protection	[108]
Sofer et al. 2013	Male adults	Genes like, *HLA-DOB, FCER1A, FCER1G, MBP, HLA-DPA1, IL-9, IL-10, ECP, and CCL11*	DNA methylation	Exposures to black carbon and sulfate	Asthma	[84]
Gaffin et al. 2014	Inner-city children	*ADRB2*	DNA methylation	-	Decreased asthma severity	[92]
Wang et al. 2015	Children	*AR, TNF-α*, and *IL-4*	DNA methylation	Phthalate exposure	Childhood asthma	[89]
Yang et al. 2015	inner-city children	*IL13, RUNX3, ST2, and IL4*	Hypomethylation and hypermethylation	-	persistent childhood atopic asthma	[91]
Sarnowski et al. 2016	Families with different nationalities	*MTNR1A*	DNA methylation	-	Co-occurrence of asthma and rhinitis	[79]
Somineni et al. 2016	Children (siblings) with age 5–18	*TET1*	DNA methylation	Exposure traffic-related air pollution	Childhood asthma	[87]
Jung et al. 2017	Children	*IL-4, IFN-γ, NOS2A, and ARG2*	DNA methylation	Short-term exposure to PM2.5 and vanadium	Childhood asthma	[81]
Chan et al. 2017	Children	-	Higher global DNA methylation	Socioeconomic status and race/ethnicity	Childhood asthma	[95]
Montrose et al. 2017	Children	*IFN-γ*	DNA methylation	Dietary intake	Childhood asthma	[98]
Wang et al. 2017	Children	*IL4*	Increased DNA methylation	Dust mite allergen-specific immunotherapy	Childhood allergic asthma	[110]
Christensen et al. 2017	C57BL6 mice	*IL-4, IL-5, IL-13, IFN-γ,* and *FOXP3*	DNA methylation	Prenatal tobacco smoke exposure	Susceptibility to asthma	[75]
Jung et al. 2017	Children	*IL-4* and *NOS2A*	Demethylation of DNA	Exposure to black carbon	Allergic asthma	[85]
Pech et al. 2018	Children	*BAT3, NEU1*, and 14 other CpGs	DNA methylation	Rhinovirus infection	Childhood asthma	[100]
Gao et al. 2018	Newborns and children	*AXL*	Hypermethylation	prenatal tobacco smoke exposure	Childhood asthma	[76]
Zhang et al. 2018	Children (siblings) with age 5–18	*NLRP3, OR2B11,* and *TET1*	DNA methylation	Traffic-related air pollution and house dust mite	Childhood asthma	[86]
Prunicki et al. 2018	Children	*Foxp3* and *IL-10*	DNA methylation	Exposure to NO2, CO, and PM2.5	Childhood asthma	[82]
Zhou et al. 2019	BALB/c mice	*IL-4*	Hyperacetylation of H3Lys9 and H3Lys14	Exposure to PM2.5 and cold stress	Asthma exacerbation	[83]
Neophytou et al. 2019	Children	*AHRR*	DNA methylation	Maternal smoking during pregnancy	Asthma development	[77]
Wu et al. 2019	Children and newborns	LMO2, GSTM1, and IL-10	Hypermethylation	Prenatal exposure to paternal tobacco smoke	Childhood asthma at age 6	[78]
Chen et al. 2019	C57BL/6 J mice	Th2 cytokine locus	Hypomethylation	Early-life undernutrition	Susceptibility to asthma	[97]
Kyburz et al. 2019	C57BL/6 mice	*CXCR3* and *FOXP3*	DNA methylation	Transmaternal Helicobacter pylori exposure	Asthma protection	[109]
Qi et al. 2020	Children	60 CpG regions associated with asthma/rhinitis	CpG methylation	active and passive smoking, molds, and pets	Asthma, rhinitis, asthma/rhinitis at age 16	[96]
Nafea et al. 2020	Children	*ADRB2*	Hypermethylation	Blood aluminum concentration	Bronchial asthma	[94]
Yang et al. 2020	Children	*MAPK1*	Hypomethylation	Bisphenol A exposure	Asthma	[90]

## Asthma development later in life and the next generations

In the previous section, we discussed how external factors could cause epigenetic changes and thus provoke asthma in individuals. These changes that seem to stem from prenatal and postnatal exposures are heritable, and they can dispose to different diseases such as asthma [112]. These epigenetic alternations in early life can provide useful information about the propensity for childhood and adulthood asthma. Besides, studies have shown that these changes may also persist into subsequent generations, also disposing them of the disease [112].

### Reflection of propensity to asthma in the epigenetic pattern

Studies have shown that it could be possible to detect the tendency to childhood or adulthood asthma in early life via the epigenetic pattern associated with immune and pro-inflammatory pathways. Augmented methylation of *SMAD3* in cord blood mononuclear cells of newborns, which was associated with the inflammatory regulator IL-1b, determined whether or not the subjects, all with asthmatic mothers, would develop asthma by age 9 [113]. These epigenetic changes can be better observed in monozygotic twin studies as they maintain the same genome but acquire a diverse epigenome. As twins grow older, they become easier to be discriminated by their epigenome from which their phenotypic differences can result. In monozygotic twins who were discordant in childhood asthma at age 10, the upstream region of *HGSNAT* gene was differentially methylated in buccal cells, whereas after eight years follow-up methylation pattern of *HLX* gene was associated with maintained asthma discordance at age 18 [114]. These findings suggest that while some gene methylation patterns are mitigated through time, some persist into adulthood. Yue et al. [115] showed that exposing pregnant mice to NO2 stimulated inflammatory responses through demethylation of the *IL-4* gene and Th2 differentiation in offsprings, which yielded adulthood allergic asthma. Likewise, a recent meta-analysis study has shown that methylation alternations of genes related to lung function that was detectible at birth in cord blood samples can bring about asthma development or other obstructive diseases in adulthood [116]. In an epigenome-wide association study, adolescent asthma was also shown to be predictable by early-life evaluation of the methylation at one CpG site within the *HK1* gene and its expression in cord blood [117]. Therefore, early-life epigenetic patterns could be a sign of asthma risk later in life span, and cord blood appears to be an ideal tissue type for detection of methylation signatures at birth.

### Transgenerational effects

Prenatal exposure has also revealed its influence on later generations by increasing asthma susceptibility that can be facilitated through aberrant epigenetic modifications. Rehan et al. [118] reported that single perinatal nicotine exposure in pups was able to alter DNA methylation and histone H3 acetylation in germ and somatic cells of the F1 generation male offsprings. Additionally, the subsequent F2 generation of these pups showed increased asthma susceptibility. Further, the risk of asthma was followed in the third generation of the pregnant mice exposed to diesel exhaust particles, and the result was the persistence of the methylation marks in that generation [119]. Despite the variable susceptibility to asthma in the three subsequent generations, they had differentiated DC-derived DNA methylation in the same loci; the loci were associated with chromatin remodeling [119]. Hypermethylation and hypomethylation in different loci of dendritic cells revealed their involvement in the transmission of maternal asthma, but part of these modifications did not cooperate in provoking the asthmatic or allergic phenotype [120, 121]. Infants born to asthmatic mothers emerged to have differentiated methylation in various loci of the DNA extracted from their peripheral blood, which may predispose them to asthma later in life [122]. In other words, offsprings' methylation pattern can be influenced by their mother's phenotype during pregnancy; as a result, this may predispose their children to a higher risk of asthma. Nevertheless, not all of these methylation changes transmitted from asthmatic mothers can lead to the disease phenotype.

**Fig. 4:**
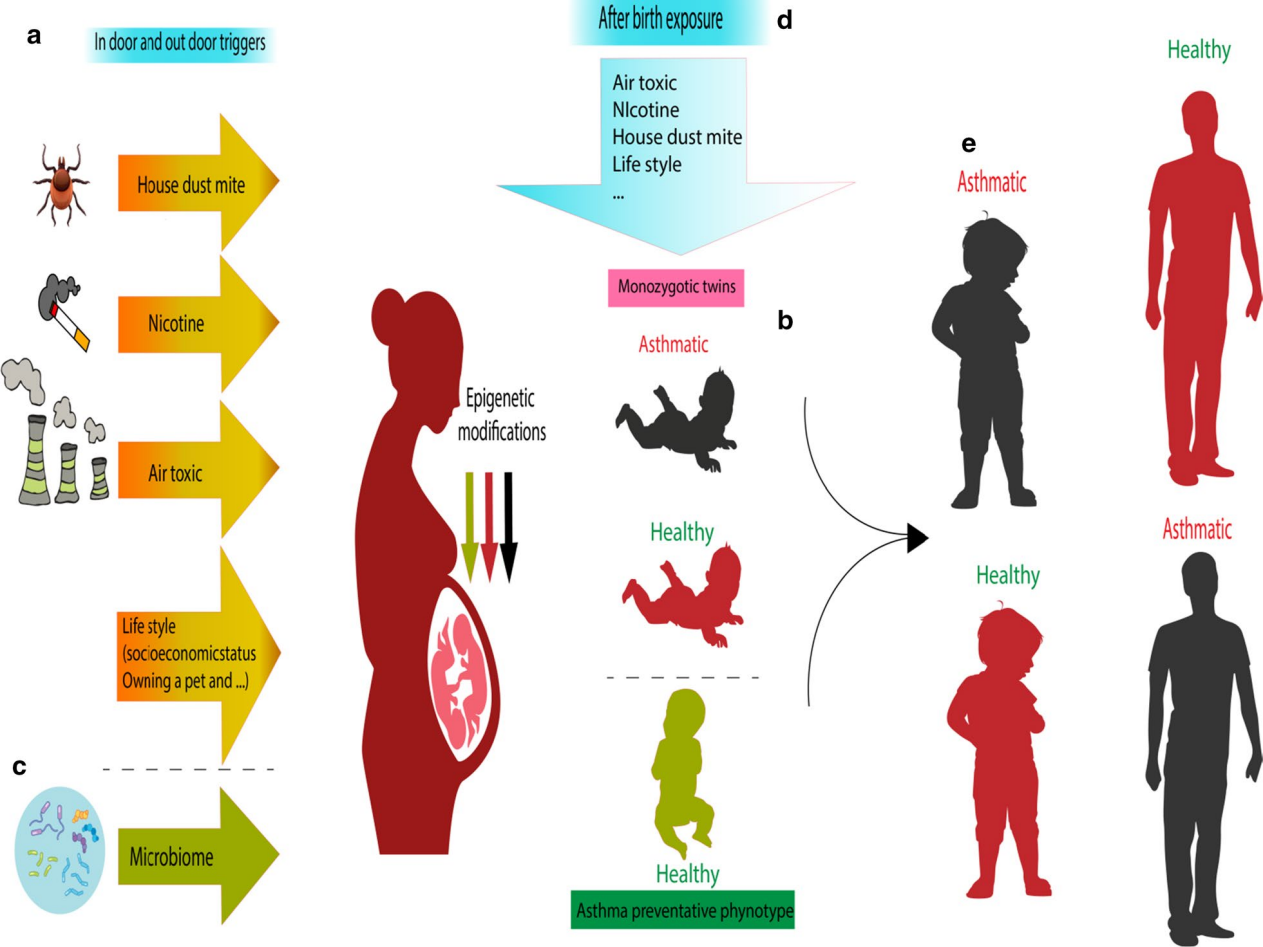
Pre- and postnatal exposure to epigenetic-mediated asthma stimulators and their outcomes. **a** Asthma triggers comprising tobacco smoking, house dust mite, air pollution, and lifestyle can negatively regulate the fetus epigenome. **b** Monozygotic twins that were exposed to these triggers in utero may or may not develop childhood asthma. **c** The microbiome may develop a protective epigenome in the offspring against asthma. **d** External factors can affect the person after birth. **e** The epigenetic changes seen in early life may affect the phenotype in childhood or adulthood, and those who showed the symptoms in childhood may recover in adulthood while those who were healthy children may become adults with asthmatic symptoms

In essence, the methylation patterns after birth may mirror the changes in the shared environment between the infant and the mother during pregnancy. Also, methylation changes in the early stages of life after birth may provoke asthma in childhood and adulthood, and there is a probability of persistence of these modified methylation patterns into the next generations. However, there were only limited in vivo experiments on the inheritance of methylation markers to the next generations, and no human research has been carried out. The overall environmental effects on pregnant mothers and their offsprings are illustrated in Fig. 4. Table 5 presents a summary of the studies in this section.

**Table 5:** Summary of the research works about the role of epigenetics in determining the propensity for asthma later in life and in the next generations

Author and year	Subject	Gene/region	Epigenetic changes	Phenotype	References
DeVries et al. 2015	Children	*SMAD3*	Hypermethylation	Childhood asthma	[113]
Murphy et al. 2015	Monozygotic twins with asthma discordancy at age 10	*HLX* and *HGSNAT*	DNA methylation	Childhood asthma discordancy	[114]
Yue et al. 2017	Pregnant BALB/C mice	*IL-4*	Demethylation of the DNA	Allergic asthma in offsprings	[115]
Everson et al. 2019	Children up to 18	*HK1*	Hypomethylation	Adolescent asthma	[117]
Rehan et al. 2012	Pregnant Sprague Dawley F0 rats	*PPARγ*	DNA methylation and histone 3 and 4 acetylation in the lung and gonad	Childhood asthma predisposition in the F2 generation	[118]
Gunawardhana et al. 2014	12-month-old infants	11 genes including *MAPK8IP3, AURKA, and PM20D1*	DNA methylation	Asthma development	[122]
Gregory et al. 2017	Pregnant BALB/C mice	41 loci	DNA methylation	Asthma susceptibility in the F2 and F3 generations	[119]
Den Dekker et al. 2019	Children	18 asthma-related differentially methylated regions	DNA methylation	Childhood asthma	[116]

## SNPs and methylation interaction

Single-nucleotide polymorphisms (SNPs) can be influenced by environmental exposure, which may provoke different human diseases [123], including asthma. Therefore, several studies have presented the interplay of SNPs and DNA methylation on the pathogenesis of asthma. Mediation analysis of asthma-related SNPs identified through genome-wide analysis studies (GWAS) demonstrated that DNA methylation mediation accounted for about 47 percent of differentiated expression of SNP-associated distant cis-genes in nasal epithelial cells, which were correlated to childhood atopic asthma [124]. A considerable number of asthma-related SNPs were located on Th2 cells enhancers, a region in which the highest rate of H3K4 dimethylation was measured in primary human CD4 + T-cells [38]. Of note, the higher the degree of H3K4 methylation, the higher the odds of H3 acetylation and inhibition of DNA methylation whereby the activation of gene transcription is ensured [125, 126]. Besides, Zhang et al. [62] demonstrated that the interactions between SNPs and DNA methylation of CpG regions in Th2 pathway genes (*IL-4, IL-4R, IL13, STAT6*, and *GATA3*) in peripheral blood leucocytes were concordant with asthma risk but the results varied in different time points. In another research on a whole-population birth cohort, only one SNP was found correlated to one CpG region of the *IL-4 receptor* gene (*IL-4R*) whose methylation levels in whole blood of 18-year-old females showed considerable association with the risk of asthma [13]. SNPs of *Interleukin-1 receptor–like 1* (*IL1RL1*), a gene that has been found associated with asthma in GWAS, contributed to the regulation of *IL1RL1* methylation in whole blood of asthmatic subjects at age 4. However, there was no considerable link between serum IL1RL1-a protein levels and methylation degree or asthma pathogenesis [127]. Likewise, the study by Larouche et al. [42] supports these results, as they also found no association between asthma and epigenetic regulation of *IL1RL1*.

The association of the SNPs of genes other than Th cells is also investigated and can be effective in the asthma pathogenesis process. Mukherjee et al. [128] by analyzing a followed-up birth cohort of subjects indicated that SNP-DNA methylation cooperation in modifying the *leptin (LEP)* gene affects the lung function and can increase the risk of asthma in peripheral blood of 18-year-old subjects. In a clinical study, in response to steroid treatment, the methylation of several groups of CpG sites on the *OTX2* gene was in concert with SNPs in nasal epithelial cells of asthmatic children, and this methylation differentiation whether dependent or independent from SNPs could distinguish good responders and poor responders to treatment [129]. Nevertheless, in childhood asthma, although the contribution of both CpG and SNPs in the *ORMDL3* expression in whole blood was observed, SNP-CpG interaction did not cooperate in this regulation [130]. On the other hand, another study revealed that the SNP at the enhancer locus of *ORMDL3* affected its CpG methylation in isolated endobronchial airway epithelial cells and, therefore, altered the gene expression in asthmatic subjects [131]. By utilizing a novel statistical method, which is canonical analysis of set interactions (CASI) on a genome-wide scale, Kogan et al. [132] have assessed statistical SNP-CpG interactions. Their analyses in DNA extracted from whole blood led to identifying three genes whose CpG sites methylation was correlated to a DNA variation in SNPs, which may be the cause of asthma pathogenesis.

To sum up, these studies emphasize the more effective function of considering both genetic and epigenetic factors rather than just one. In this section, most of the studies were conducted on DNA extracted from whole blood and PBMC and very few on epithelial cells. Unlike nasal samples, there is a mixture of epigenetic patterns in whole blood and PBMC due to the presence of a variety of cells, so they cannot be considered an ideal tissue type. Importantly, it is revealed that the link between CpG methylation and SNPs depends on the time points, so it highlighted the key role of age in this area of study, which is poorly investigated. Table 6 presents a summary of experiments in which the interaction between SNPs and DNA methylation is assessed.

**Table 6:** Summary of the research works about SNP-CpG methylation interaction on specific genes in asthma

Author and year	Subjects	Specimen	Significant SNP/CpG	Gene	References
Soto-Ramírez et al. 2013	18-year-old females	Whole blood	rs3024685/ cg09791102	*IL4R*	[13]
Zhang et al. 2014	Girls with the age of 10 and 18	Whole and peripheral blood	rs3024685, rs8832/ cg26937798 (Related to IL4R)	IL4, IL4R, IL13, GATA3, and STAT6	[62]
Acevedo et al. 2015	Boys and girls at age 8	whole blood	rs7216389, rs4065275 and rs12603332	*GSDMB* and *ORMDL3*	[130]
Mukherjee et al. 2016	18-year-old men and women and a subset of women at age 10	Peripheral blood	rs11763517/cg00666422	*LEP*	[128]
Nicodemus-Johnson et al. 2016	Adults	Endobronchial airway epithelial cells	rs2517955/cg05616858	*ORMDL3*	[131]
Zhang et al. 2017	Children with ages 5–8	Nasal epithelial cells	6 significant interaction sites out of 182 SNPs	*OTX2* and *LDHC*	[129]
Dijk et al. 2018	Children at age 4	Whole blood	rs1420101/cg11916609, rs56179005/cg20060108,rs76886731/cg25869196,and rs1420104/cg19795292	*IL1RL1*	[127]
Kim et al. 2019	Puerto Rican and African-American children	Nasal epithelial cells	-	*-*	[124]
Kogan et al. 2019	Adults	Whole blood	rs10818651/cg21469772, rs10985567/cg21213617	*LOC101928523, SCARNA18, LHX6, and STC1*	[132]

## Epigenetic differentiation studies

Further implications of identifying epigenetic differentiation in asthma are to discern types and stages of asthma, advantages, and disadvantages of treatment and also to discover a suitable tissue type for these studies. The methylation profile of asthma remission, despite the absence of asthma symptoms, was distinguishable from healthy controls by the difference in 1163 CpG sites and 328 regions in bronchial biopsies. Additionally, remission and persistence in asthma varied by even a smaller difference, 42 regions [133]. Among the numbers of loci globally differentiating allergic subjects, aspirin-intolerant asthmatic subjects, and control group, the *CYP26A1* gene was distinctly methylated in each of them. Hypermethylation of *the CYP26A1* promoter and decrease in its expression was observed in the PBMC isolated CD19 + B lymphocytes of the cohort with allergic asthma utilizing HELP assay [134]. Gunawardhana et al. [135] identified PBMC CpG loci differentially methylated in three inflammatory phenotypes of asthma assessed in CD14 + purified monocytes, including eosinophilic asthma, paucigranulocytic asthma, and neutrophilic asthma. Their results also suggested that a distinct type of asthma may result from an aberrant cytokine expression that may be the result of varied methylation patterns. Studying discordancy in monozygotic twins about ex vivo data, Runyon et al. [136] inferred that the varied DNA methylation patterns of asthmatic adults were associated with the dysfunction of effector T-cells (Teff) and Tregs isolated from whole peripheral blood and BALF. This study also demonstrated that increased methylation of *FOXP3* and *IFN-γ* led to their lower expression in Tregs and Teffs, respectively, in discordant asthmatic twin pairs.

**Fig. 5:**
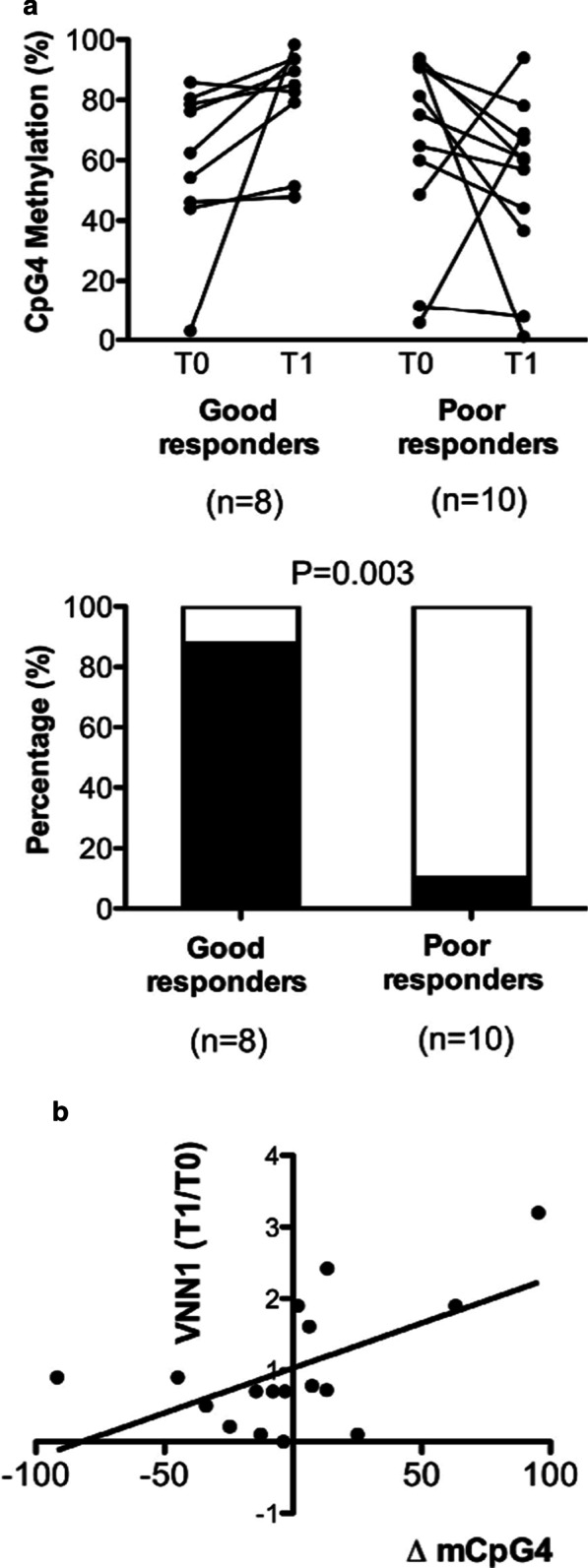
Change in DNA methylation and mRNA expression of *Vanin-1* (*VNN1*) in response to corticosteroid treatment. **a** The differentiation of CpG methylation ratio in *VNN1* promoter region between good responders and bad responders to corticosteroid treatment. **b** the association between the *VNN1* CpG methylation and the mRNA expression [Reprinted with permission from Elsevier] [141]

Several investigations have been done signifying the correlation between asthma severity and methylation levels of asthma-related genes. In 2011, Isidoro-Garcı´a et al. [137] found different levels of methylation at the *prostaglandin D2 receptor* gene (*PTGDR*) promoter in various asthmatic patients. Their results demonstrated that the hypomethylation of the *PTGDR* gene provoked an increase in its expression in PBMC derived CD19 + B lymphocytes. This led to a hypothesis that the different methylation level was related to the severity of allergic asthma, thus making it a future therapeutic and diagnostic target. Nevertheless, although *ADAM 33* is identified as a factor underlying asthma exacerbation [138], there was not a considerable difference in peripheral blood leukocytes CpG methylation of the exon 9 of this gene between asthmatic adults and controls [139]. In a study by Cosio et al. [140], both mild and severe asthma patients witnessed declined HDAC and augmented HAT activities in PBMC derived alveolar macrophages. The results show that glucocorticoid treatment in patients with moderate asthma results in HDAC1 increase and restrain TNF-α and HAT and, therefore, suppresses Nf-kB and through that restrains IL-8. Only mild asthmatic patients responded to prednisolone treatment with increased HDAC1 and decreased IL-8 [140]. In another study using nasal epithelial cells, effective or feeble response to corticosteroid treatment was reflected in DNA methylation of the *Vanin-1* gene promoter as well as the mRNA expression in childhood asthma (Fig. 5) [141]. Furthermore, genome-wide studies identified several CpG regions on the *OTX2* gene whose DNA methylation decrement in nasal epithelial cells was considered a response to steroid treatment in well-responded children with asthma [129]. Moreover, severe eosinophilic asthma with glucocorticoid resistance showed a lower level of HDAC2 and total HDAC than that of moderate patients [37]. These studies suggest that asthma severity depends on the methylation or acetylation degree, but these modifications are highly dependent on the cell type, and as long as characteristic dependent effects, like age and gender, on them are not precluded, these sorts of measurements may be inconclusive and biased.

Reflection of response to the treatments differs in mild and severe asthma as well as in different tissue types. Regarding the deductions of several studies, PBMC is not a well-recommended tissue type to study the epigenetic process of asthma [47, 49, 140, 142] as opposed to airway epithelial cells and nasal epithelial cells [143]. An epigenome-wide association study identified associations between asthmatic phenotype and methylation profile in different CpG sites using peripheral blood leukocytes, indicating that using whole blood instead can bias the results [143]. On the contrary, Xu et al. [144] via a large-scale epigenome-wide meta-analysis have shown that whole blood methylation analysis can be beneficial in finding differentiated CpG methylation correlated to childhood asthma, and since their findings were also partially in line with airway epithelial cells. In their study, 14 CpG regions were identified showing consistent hypomethylation in different time points, while it was not the case for cord blood DNA [144]. Moreover, another epigenome-wide meta-analysis study has also revealed new CpG sites with differentiated methylation in cord blood and whole blood of newborns and children with asthma, respectively, with only the latter being replicable in respiratory epithelium cells and eosinophils [145]. Yang et al. [146] in a study on childhood atopic asthma in 2017, compared their discovered methylation changes in nasal epithelial cells with their findings derived from PBMC in their previous study in 2015. Although the differentiated methylation profiles had few overlaps between PBMC and epithelial cells, findings from the latter were about tenfold greater than the former. Further, through recent bioinformatics analysis, Shi et al. [147] have found 146 differentially methylated genes, including *tumor necrosis factor* (*TNF*) and *HLA-DBA1*, using the data from the study by Yang et al. [146] in which 186 differentially methylated genes were discovered in nasal epithelial cells in children with atopic asthmatic. In both of these studies, methylation alternations in genes were concordant with their expression. This also shows that bioinformatics is a viable approach to analyze methylation modifications and their consequent impact on expression in asthma and epithelial cells.

In brief, a positive point about these studies is that almost all of them were cell-specific, which increases the reliability of their outcomes. There was no in vivo experiment on this aspect, and all of them were human research. In both histone modification and DNA methylation studies, whole blood or PBMC did not perform as an ideal type of tissue, although PBMC was used in most of the cell-specific studies. Buccal cells have also shown potential for measuring epigenetic modifications and it is more advantageous in sampling children as it is less invasive than others. However, Talens et al. [148] suggested that there is a correlation between methylation in blood and buccal cells, so it cannot be fully determined which one is more suitable. As sampling bronchial epithelium is not readily possible, nasal epithelial cells seem to be a suitable surrogate for being safer and easier to obtain and can also be a reflection of the changes in bronchial airway cells [149, 150]. Table 7 presents a list of studies that are reviewed in this chapter.

**Table 7:** Summary of epigenetic differentiation studies in asthma

Author and year	Subjects	Specimen	Status	Gene/region	Differentiation	References
Cosio et al. 2004	Adults with atopic asthma	Alveolar macrophages and peripheral blood monocytes	Mild, moderate, and severe asthma in response to drugs that change HDAC and HAT activity	*HDAC*	Histone acetylation	[140]
Pascual et al. 2011	Adults with allergic asthma	PBMC	allergic subjects, aspirin-intolerant asthmatics, and healthy subjects	*CYP26A1*	Hypermethylation	[134]
Isidoro-García et al. 2011	Adults with allergic asthma	PBMC derived CD19 + B lymphocytes	Asthma severity	*PTGDR*	Hypomethylation	[137]
Runyon et al. 2012	Adult monozygotic twins	Peripheral whole blood and BALF	Childhood asthma discordancy	*IFN-γ and FOXP3*	Hypermethylation	[136]
Yang et al. 2013	Adults	Peripheral blood leukocytes	Asthma severity	*ADAM33*	Same methylation pattern	[139]
Li et al. 2013	Adults with allergic asthma	Peripheral blood T-cells	Severe eosinophilic asthma with glucocorticoid resistance	*LAT*	Histone hypoacetylation	[37]
Gunawardhana et al. 2014	Male and female adults with stable asthma	Peripheral blood CD14 + monocytes	eosinophilic asthma, paucigranulocytic asthma, and neutrophilic asthma	223, 237, and 72 differentially methylated CpG loci	Hypermethylation	[135]
Xiao et al. 2015	Children	Nasal epithelial cells	Corticosteroid treatment response	*Vanin-1*	DNA methylation	[141]
Zhang et al. 2017	Children	Nasal epithelial cells	Steroid treatment response	*OTX2* and *LDHC*	DNA methylation	[129]
Yang et al. 2017	Inner-city children	Nasal epithelial cells	Persistent atopic asthma	186 genes	DNA methylation	[146]
Xu et al. 2018	Children	Nasal cells, cord blood, whole blood, peripheral blood	Allergic asthma	14 CpG Sites	Hypomethylation	[144]
Reese et al. 2019	Newborns and children	Whole blood and nasal respiratory epithelium cells and eosinophils	Asthma diagnosis in newborns and children	179 CpGs with 36 regions in children and 9 CpGs with 35 regions in newborns	DNA methylation	[145]
Shi et al. 2020	Children	Nasal epithelial cells	Childhood atopic asthma	*TNF and HLA-DBA1*	DNA methylation	[147]
Vermeulen et al. 2020	Adults	Ciliated epithelial cells, basal cells, smooth muscle cells, fibroblasts, neutrophils, and T-cells	Persistence and remission in asthma and in different cell types	4 differentially methylated CpG sites and 42 regions	DNA methylation	(133)

## Concluding remarks and suggestions for future works

This article has explained the modulation of asthma predisposition, pathogenesis, and exacerbation through epigenetic modifications. DNA methylation is the most studied area in asthma, playing a key role in mediating environmental effects and interactions with genetic elements. Although histone modifications contribute to the oxidative stress process caused by environmental factors, only a small number of studies are allocated to the role of these modifications caused by various external stimuli. What is more, epigenetic changes, including both asthma inhibitors and developers, can be passed on to future generations. Since these changes are reversible, improving our knowledge in this area can lead to discovering efficient therapeutic targets and drugs as well as diagnostic tools for different types of asthma. Additionally, due to the existing cross talk between histone modifications and other epigenetic regulations [151], exploring the combined effects of them in regulatory genes in asthma may provide valuable information. Several studies mentioned the potential effect of owning pets as early-life exposures on methylation patterns and in the development of asthma, but it is not yet investigated exclusively. Notably, since the majority of studies were carried out after the disease occurrence, it cannot yet be determined whether epigenetic effects caused inflammation in asthma or the opposite, but longitudinal studies certainly pave the way for elucidating this matter. Although followed-up birth cohort studies were able to estimate the odds of asthma before the disease occurrence based on methylation differentiation, these studies lacked sample population size or some of them failed to replicate their results; therefore, more studies need to be done in this area. Several factors influenced most of the studies. The studies that assessed both SNPs and DNA methylation found the link between these two vital when measuring methylation differentiations. Age was also another pivotal factor that affected the results of almost all of the studies on epigenetics in asthma. The type of tissue used for the study highly influences the results because not only epigenetic regulations are cell-specific but also tissues like nasal epithelial cells can reflect the changes in the epithelium better than tissues like whole blood or PBMC. Each future study should consider more of these factors simultaneously when analyzing epigenetic modifications involved in asthma.

## Data Availability

Not applicable.

## Abbreviations

ARG: Arginase
ADRB2: The beta-2 adrenergic receptor
BMI: Body mass index
BALF: Bronchial alveolar fluid
CpG: Cytosine-phosphate-guanine
FeNO: The fractional concentration of NO
FOXP3: Forkhead box P3
GWAS: Genome-wide analysis studies
HDAC: Histone deacetylases
HAT: Histone acetyltransferase
IFN-γ: Interferon-gamma
IL: Interleukin
iNOS: Induced nitric oxide synthase
LEP: Leptin gene
NO: Nitric oxide
Nf-kB: Nuclear factor-κB
PTGDR: Prostaglandin D2 receptor
PBMC: Peripheral blood mononuclear cells
SNP: Single-nucleotide polymorphisms
Socs3: Suppressor of cytokine signaling-3
TNF: Tumor necrosis factor
Th: T helper cells
Teff: Effector T-cells
Treg: Regulatory T-cells
